# BRCA1 and homologous recombination: implications from mouse embryonic development

**DOI:** 10.1186/s13578-020-00412-4

**Published:** 2020-03-30

**Authors:** Yidan Liu, Lin-Yu Lu

**Affiliations:** 1 Key Laboratory of Reproductive Genetics (Ministry of Education) and Women’s Reproductive Health Laboratory of Zhejiang Province, Women’s Hospital, Zhejiang University School of Medicine, Hangzhou, China; 2 Institute of Translational Medicine, Zhejiang University School of Medicine, Hangzhou, China

## Abstract

As an important player in DNA damage response, BRCA1 maintains genomic stability and suppresses tumorigenesis by promoting DNA double-strand break (DSB) repair through homologous recombination (HR). Since the cloning of *BRCA1* gene, many *Brca1* mutant alleles have been generated in mice. Mice carrying homozygous *Brca1* mutant alleles are embryonic lethal, suggesting that BRCA1’s functions are important for embryonic development. Studies of embryonic development in *Brca1* mutant mice not only reveal the physiological significance of BRCA1’s known function in HR, but also lead to the discovery of BRCA1’s new function in HR: regulation of DSB repair pathway choice.

## Introduction

*BRCA1* is a well-known breast and ovarian cancer susceptible gene that is frequently mutated in familial breast and ovarian cancers [1]. *BRCA1* mutation carriers also have increased risk of other cancers such as pancreatic cancer and prostate cancer [2, 3]. Since the cloning of the *BRCA1* gene more than two decades ago [4], the functions of BRCA1 have been extensively studied. Despite participating in multiple cellular processes, BRCA1 is most well-characterized for its functions in DNA damage response. BRCA1 translocates to DNA damage sites and coordinates both DNA damage repair and DNA damage signaling [5], which are essential for maintaining genomic stability and suppressing tumor formation [6, 7].

DNA double-strand break (DSB) is the most deleterious form of DNA damage that can be generated by exogenous DNA damaging agents or endogenous replication stress. DSBs can be repaired by homologous recombination (HR) or non-homologous end joining (NHEJ). BRCA1 functions in multiple steps to promote DSB repair by HR [8]. BRCA1-deficient cells are HR-deficient and are sensitive to DSB-inducing agents such as platinum-based drugs. Subsequent studies have found that BRCA1-deficient cells are ultra-sensitive to PARP inhibitors (PARPi) [9]. PARP inhibition blocks base excision repair and results in conversion of DNA single-strand breaks (SSBs) to DSBs. PARPi also trap PARP1 on chromatin that requires fixation by HR repair. Therefore, PARPi specifically kill HR-deficient cells, such as BRCA1-deficient cells [10]. PARPi have achieved great success in preclinical mouse models as well as in clinical trials to treat BRCA1-deficient cancers [11]. As a result, several PARPi have been approved for clinical use. However, PARPi resistance has developed over time in many cancer patients, in part by restoring HR [12]. Uncovering the mechanisms how HR is restored in these patients is essential for developing strategies to overcome PARPi resistance.

Studies from the past decade have revealed that BRCA1 promotes HR repair of DSBs at multiple stages. At DSB sites, BRCA1 regulates DSB repair pathway choice by promoting HR over NHEJ, which is achieved by countering 53BP1’s block at DSB ends and promoting DNA end resection, a pre-requisite and determinant step for HR [13–15]. After generation of single-strand DNA (ssDNA) by DNA end resection, BRCA1 directly interacts with PALB2 and recruits BRCA2/RAD51 to DSB sites to form RAD51-ssDNA filaments for strand invasion [16–18]. A recent study reveals that BRCA1, together with its dimerization partner BARD1, enhances the recombinase activity of RAD51 and promotes RAD51-mediated pairing of homologous sequences [19]. Collectively, BRCA1 functions at three key steps of HR repair of DSBs [8]. In addition, BRCA1 stabilizes stalled replication forks and prevents them from collapsing after replication stress [20]. BRCA1 also promotes the cleavage-coupled break-induced replication pathway to restart stalled replication forks [21]. These functions of BRCA1 can decrease the incidences of DSB formation after replication stress.

## Overview of *Brca1* mutant mice

Our current knowledge about BRCA1’s function in HR repair is obtained through numerous studies using multiple approaches, including biochemical assays, molecular and cellular studies, crystallography, human genetics, as well as mouse genetics. When *BRCA1* gene was first cloned [4], techniques for generating gene knock-out and knock-in mice has become routine. Since then, many mutant alleles of *Brca1* have been generated in mice [22]. Characterization of these *Brca1* mutant mice has contributed tremendously to the understanding of the physiological functions of BRCA1, especially their roles in HR repair.

To date, more than 20 *Brca1* mutant alleles have been generated in mice, including mutations, deletions, conditional deletions, and alleles with humanized sequence. Mice homozygous for many *Brca1* mutant alleles are embryonic lethal, suggesting that BRCA1 is important for embryonic development. Conditional knockout of *Brca1* in specific tissues have revealed that BRCA1 is important for the development of breast, heart, brain, hair, testis, lymphocytes, and other organs [22]. Tissue-specific *Brca1* knockout or mutations, in combination with other transgene or gene knockout, have also been generated for studying BRCA1’s role in the development of breast, ovarian, and pancreatic cancers [22, 23]. Supporting the observations in human patients, most *Brca1* knockout and mutations accelerate tissue-specific cancer development in mice [22, 23].

The large number of *Brca1* mutant alleles generated reflects the difficulty of understanding BRCA1’s functions by interpreting mouse phenotypes of individual mutant alleles. For example, although mice homozygous for many *Brca1* mutant alleles cause embryonic lethality, embryos carrying different *Brca1* mutant alleles die at different stages of development, suggesting that these alleles have different impact on BRCA1’s functions. This is largely due to the size of BRCA1 proteins, and presence of distinct functional domains, different isoforms, and various post-translationally modifications. Some *Brca1* mutant alleles can only disrupt the functions of certain domains but not the entire BRCA1 protein [22]. On the other hand, analyses of different phenotypes from different mutant alleles provide valuable information about the contribution of different sites, domains, or isoforms to the functions of this large protein, particular in HR repair [22]. In addition, studies of gene disruptions that rescue the embryonic lethality of *Brca1* mutant mice have led to the discovery of new functions of BRCA1 in HR. In this article, we will focus on the embryonic lethality phenotypes of *Brca1* mutant mice and discuss how different *Brca1* mutant alleles have contributed to our understanding of BRCA1’s function in HR repair.

## Embryonic development defects of *Brca1* mutant mice

BRCA1 is a large protein comprising several important domains. On the N-terminus is a RING domain that interacts with the RING domain of BARD1 to form a constitutive heterodimer [24–26]. The RING-RING dimer also harbors E3 ubiquitin ligase activity that is important for HR repair [27, 28]. In the middle is a large region consisting around 60% of the total amino acids of BRCA1 protein, which is encoded by a single exon 11. No specific domains except two nuclear localization signals (NLS) have been identified in this region [29, 30]. This region is followed by a coiled-coiled (CC) domain that directly interacts with PALB2 and facilitates the loading of BRCA2 and RAD51 to DSB sites [16–18]. On the C-terminus are tandem BRCT domains that recognize phosphorylated peptides and mediate the interaction of BRCA1 with several phosphorylated proteins [31–33]. These domains are required for the formation of distinct BRCA1 complexes (BRCA1-A/B/C complex) with distinct functions [34]. In this section, we will categorize all *Brca1* mutant alleles by the domains disrupted and discuss how they impact BRCA1’s function in embryonic development. A summary of all *Brca1* mutant alleles can be found in Table 1.

**Table 1:** Summary of all *Brca1* mutant alleles

MGI allele ID	Allele symbol	Synonyms	Embryonic development phenotypes (homozygous)	References
Mice with complete disruption of BRCA1				
MGI:1858108	Brca1 <tm1Mak>	*Brca1*^*Δ5-6*^	Die before E7.5	[35]
MGI:2429692	Brca1 <tm2Mak>	*Brca1*^*flox5-6*^	Conditional, viable	[38]
MGI:3762184	Brca1 <tm1.1Brn>	*Brca1*^*Δ5-13*^	Embryonic lethal, embryonic development not analyzed	[39]
MGI:3696057	Brca1 <tm1Brn>	*Brca1*^*flox5-13*^	Conditional, viable	[39]
Mice with Exon11 disruptions				
MGI:1857931	Brca1 <tm1Bhk>	*Brca1*^*Δ223-763*^	Die between E8.5 to E13.5	[40]
MGI:1930612	Brca1 <tm1Cxd>	*Brca1*^*11−*^	Die between E7.5 and E9.5	[42]
MGI:1858107	Brca1 <tm1Whl>	*Brca1*^*Δ300-361*^	Die before E7.5	[43]
MGI:2177209	Brca1 <tm2Arge>	*Brca1*^*tr*^	Largely viable, viability depends on genetic background	[44]
MGI:2182470	Brca1 <tm2.1Cxd>	*Brca1*^*Δ11*^	Seldomly viable, most die between E12.5–18.5	[45]
MGI:1931238	Brca1 <tm2Cxd>	*Brca1*^*flox11*^	Conditional, viable	[45]
MGI:3665173	Brca1 <tm4Cxd>	*Brca1*^*FL*^	Viable	[47]
Mice with RING domain disruptions				
MGI:1930613	Brca1 <tm1Arge>	*Brca1*^*ex2*^	Die between E6.5 and E9.5	[48]
MGI:3790741	Brca1 <tm1Thl>	*Brca1*^*flex2*^	Conditional, viable	[49]
MGI:5823771	Brca1 <tm2Jjon>	*Brca1*^*185stop*^	Die between E9.5 to E13.5	[50]
MGI:5307254	Brca1 <tm1.1Jjon>	*Brca1*^*C61G*^	Die between E9.5 and E12.5	[51]
MGI:5494435	Brca1 <tm3.1Thl>	*Brca1*^*I26A*^	Viable	[41]
Mice with BRCT domain disruptions				
MGI:2178447	Brca1 <tm1Rfo>	*Brca1*^*1700T*^	Die between E9.5 and E10.5	[53]
MGI:5823772	Brca1 <tm3Jjon>	*Brca1*^*5382stop*^	Die between E9.5 and E12.5	[50]
MGI:3706167	Brca1 <tm1Aash>	*Brca1*^*F22-24*^	Conditional, viable	[54]
MGI:6281370	Brca1 <em1Njo>	*Brca1*^*ΔC*^	Embryonic lethal, embryonic development not analyzed	[56]
MGI:5494434	Brca1 <tm2.1Thl>	*Brca1*^*S1598F*^	Viable	[41]
Mice with disrupted phosphorylation				
MGI:3513253	Brca1 <tm3Cxd>	*Brca1*^*S971A*^	Viable	[61]
MGI:4418211	Brca1 <tm5.1Cxd>	*Brca1*^*S1152A*^	Viable	[62]

### Mice with complete disruption of BRCA1

Among the first batches of *Brca1* mutant alleles generated, *Brca1*^*Δ5-6*^ allele is the only null allele [35]. In this allele, exon 5 and 6 are replaced by a neomycin cassette, disrupting sequences encoding the RING domain and generating stop codons in all reading frames. Experiments confirm that BRCA1 is indeed absent from embryos homozygous for *Brca1*^*Δ5-6*^ allele [35]. Mice homozygous for this allele are embryonic lethal. Homozygous embryos seem normal before implantation, but postimplantation embryos die before E7.5. Abnormal embryonic development with hindered gastrulation starts to be observed at E5.5, and there are clear defects of epiblast cell proliferation in these embryos. Consistently, inner cell mass from homozygous blastocysts do not grow in vitro and no homozygous embryonic stem cells can be obtained. Based on these observations, it is postulated that BRCA1 is required for cell proliferation. However, this hypothesis seems contradictory to the observation in *BRCA1* mutant human cancers, where *BRCA1* mutations do not compromise cell proliferation. It is possible that BRCA1 is only required for the proliferation of certain cell lineages. Since BRCA1 is required for HR repair, it is likely that HR deficiency contributes to the defects in homozygous *Brca1*^*Δ5-6*^ embryos [35]. In agreement with this possibility, loss of RAD51, the key enzyme for HR repair, leads to similar defects in mice [36, 37]. Therefore, HR repair is required for proper cell proliferation and early embryonic development in mice. *Brca1*^*flox5-6*^ is a *Brca1* conditional null allele, which is seldomly used to study the tissue-specific function of BRCA1 [38].

Another *Brca1* null allele, *Brca1*^*Δ5-13*^, has been generated from a *Brca1* conditional null allele, *Brca1*^*flox5-13*^ [39]. In this allele, sequences from exon 5 to 13 are deleted, disrupting all functional domains of BRCA1. Mice homozygous for *Brca1*^*Δ5-13*^ allele are also early embryonic lethal, but the defects in embryonic development are not characterized. Importantly, *Brca1*^*flox5-13*^ allele is a *Brca1* conditional null allele that is frequently used. This allele has been used to examine tissue-specific functions of BRCA1 and the phenotypes can fully reflect BRCA1’s functions in these tissues. This allele has also been used to generate tissue-specific BRCA1 null cancer models. Tumors from these mice should reflect the characteristics of human cancers with complete loss of *BRCA1* expression.

### Mice with Exon 11 disruptions

Exon 11 is the largest exon that spans more than half of the coding sequences of the *Brca1* gene. Besides nuclear localization signals, no domains are encoded by this exon. A number of alleles disrupting exon 11 have been generated, but there are some differences in the phenotypes observed. In the first allele, *Brca1*^*Δ223-763*^, 330 bp of intron 10 and 1.5 kb of exon 11, including the splice acceptor for exon 11, are replaced by a neomycin cassette, deleting amino acids 223–763 of BRCA1 protein [40]. Mice homozygous for this allele are embryonic lethal. The lethality occurs between E8.5 and E13.5, which is much latter than embryos homozygous for *Brca1*^*Δ5-6*^ null allele. ES cells homozygous for *Brca1*^*Δ223-763*^ allele are viable, but HR repair efficiency is severely compromised [41]. A similar allele, *Brca1*^*11−*^, is generated in which 330 bp of intron 10 and 407 bp of exon 11 are replaced by a neomycin cassette [42]. Although shorter sequences are deleted, embryos homozygous for this allele die earlier between E7.5 and E9.5. In the third allele, *Brca1*^*Δ300-361*^, an even shorter sequence within exon 11 is replaced by a neomycin cassette, deleting amino acids 300–361 of BRCA1 protein [43]. Surprisingly, although the sequence deleted in exon 11 is the shortest in *Brca1*^*Δ300-361*^ allele, embryos homozygous for this allele display much more severe phenotype. Homozygous embryos are abnormal starting from E4.5–5.5 and are dead by E7.5, which is similar to the phenotypes of homozygous *Brca1*^*Δ5-6*^ null embryos. The reasons behind these observations are not clear.

In the fourth allele, *Brca1*^*tr*^, a piece of 50 bp DNA is inserted in exon 11 and causes protein termination at amino acid 924 [44]. Non-sense mediated mRNA decay leads to dramatic reduction of full-length transcript, but the natural Δ11 isoform is still normally produced. Therefore, this allele is a hypomorphic allele. Mice homozygous for this allele are viable depending on genetic background. They are completely viable in 129/Sv or MF1 background, but the viability is dramatically reduced in C57BL/6J background. The viable homozygous mutant mice develop a variety of tumors including breast tumors.

An allele with precise exon 11 deletion, *Brca1*^*Δ11*^, is generated from a *Brca1* conditional Δ11 allele, *Brca1*^*flox11*^ [45]. As the full-length isoform (around 220 kDa) is converted to Δ11 isoform (around 100 kDa), the expression of Δ11 isoform is higher than usual. *Brca1*^*Δ11/Δ11*^ mice is largely embryonic lethal, although a very small number of mice can be found in newborn mice. *Brca1*^*Δ11/Δ11*^ embryos die between E12.5–18.5, suggesting that the full length BRCA1 is required for embryonic development. Since *Brca1*^*Δ11/Δ11*^ embryos die at a later stage than *Brca1* null embryos, it is likely that BRCA1Δ11 protein still retains some functions of BRCA1. This is not surprising because BRCA1Δ11 protein still contains the RING, CC, and BRCT domains. Despite partially defective in nuclear localization, this protein still dimerizes with BARD1, interacts with PALB2, and localizes to DNA damage sites [46]. Therefore, *Brca1*^*Δ11*^ allele is a hypomorphic allele. The conditional Δ11 allele, *Brca1*^*flox11*^, has been used in many studies to examine tissue-specific function of BRCA1 and to establish tissue-specific tumor models. Given that BRCA1’s function is partially retained in BRCA1Δ11 protein, caution should be taken when interpreting studies using this allele. It is also surprising that *Brca1*^*Δ11/Δ11*^ embryos die much later than mice homozygous for the above three alleles that disrupt exon 11, which implies that the above three alleles not only disrupt exon 11 but also affect other regions of *Brca1* gene.

*Brca1Δ11* transcript isoform is naturally present, but it is not clear if this isoform has specific functions. In order to address this issue, the *Δ11* isoform is specifically disrupted without affecting the full-length isoform [47], creating a full-length isoform-only allele *Brca1*^*FL*^. Mice homozygous for this allele are fully viable. They have no obvious phenotypes except for elevated tumor formation, uterine hyperplasia, and mammary gland abnormalities. Therefore, the *Δ11* isoform is dispensable for mouse development but might be required for some subtle functions of BRCA1 in older mice.

### Mice with RING domain disruptions

BRCA1 is a big protein with several functional domains. Although complete gene disruption in mice reveals that BRCA1 is required for embryonic development, disruption of individual domains in mice can provide additional insights into the mechanism underlying BRCA1’s functions in this process. In addition, disruption of individual domains in mice can mimic the mutations in cancer patients so that these mice can be used for investigating the role of individual domains in tumor suppression. Currently, several alleles have been generated to disrupt the RING and the BRCT domains of BRCA1 in mice.

The RING domain of BRCA1 interacts with BARD1 to form a heterodimeric E3 ubiquitin ligase. The first allele disrupting the RING domain is *Brca1*^*ex2*^, in which a neomycin cassette replaces exon 2 that encodes part of the RING domain [48]. Since the translation start codon is present in exon 2, it is believed that BRCA1 translation is abolished so that *Brca1*^*ex2*^ allele is a null allele. Mice homozygous for *Brca1*^*ex2*^ allele are embryonic lethal, but lethality occurs from E6.5 to E9.5, which is later than *Brca1* null mice *Brca1*^*Δ5-6/Δ5-6*^. Recently, it is found that disruption of exon 2 in *Brca1*^*ex2*^ allele generates a transcript in which exon 1 is directly spliced to exon 3. Although the original translation start codon in exon 2 is deleted, another translation start codon is activated at Met-90 or Met-99 to produce a truncated BRCA1 protein that lacks most amino acids of the RING domain but retains the amino acids for the rest of the protein. Therefore, *Brca1*^*ex2*^ allele is not a null allele but a mutant allele that produces a BRCA1ΔRING protein, which explains the different embryonic development defects between *Brca1*^*ex2/ex2*^ and *Brca1*^*Δ5-6/Δ5-6*^ mice. A conditional allele with precise deletion of exon 2, *Brca1*^*flex2*^, has also been generated [49]. Mice carrying *Brca1*^*ex2*^ and *Brca1*^*flex2*^ alleles have been used as the null and conditional null allele in several studies. Therefore, caution should be taken when interpreting the results of these studies.

A similar allele, *Brca1*^*185stop*^, is generated in mice to mimic the common founder mutation *BRCA1*^*185delAG*^ in human cancer patients [50]. Similar to *Brca1*^*ex2*^ allele, *Brca1*^*185stop*^ allele causes translation to start from a downstream start codon at Met-90 and produces a BRCA1ΔRING protein. Mice homozygous for *Brca1*^*185stop*^ allele are embryonic lethal and embryos die between E9.5 to E13.5. The third allele, *Brca1*^*C61G*^, is generated in mice to mimic one of the most frequent missense mutations, *BRCA1*^*C61G*^, in human cancer patients [51]. Instead of generating stop codons and producing a BRCA1ΔRING protein, this mutation disrupts the structure of the RING domain, reduces BARD1 binding, and abolishes the E3 ubiquitin ligase activity. Similar to *Brca1*^*185stop*^ allele, embryos homozygous for *Brca1*^*C61G*^ allele die between E9.5 and E12.5.

Instead of disrupting the RING domain structure, a point mutation is generated in *Brca1*^*I26A*^ allele to specifically abolish the E3 ligase activity of BRCA1 [41]. Interestingly, mice homozygous for *Brca1*^*I26A*^ allele is viable, suggesting that the E3 ligase activity of BRCA1 is dispensable for embryonic development. In agreement with this observation, abolishing the E3 ubiquitin ligase activity of BRCA1 does not affect cell viability or HR repair either [52]. It seems that the structural role, but not the catalytic role, of the RING domain is important for BRCA1’s function in HR repair and embryonic development.

### Mice with BRCT domain disruptions

The tandem BRCT domains on the C-terminus of BRCA1 interact with multiple phosphorylated proteins and are required for BRCA1’s localization at DSB sites. The first allele disrupting the tandem BRCT domains, *Brca1*^*1700T*^, is generated by inserting a neomycin cassette into exon 20 to remove the last BRCT domain [53]. Mice homozygous for this allele are embryonic lethal and homozygous embryos die between E9.5 and E10.5, which is less severe than *Brca1* null embryos. The second allele, *Brca1*^*5382stop*^, is generated to mimic the common founder mutation *BRCA1*^*5382insC*^ in human cancer patients, which leads to deletion of the last BRCT domain as well [50]. Similarly, homozygous embryos for this allele die between E9.5 and E12.5, which is less severe than *Brca1* null embryos. A conditional allele, *Brca1*^*F22-24*^, is also generated that deletes exons 22 to 24 and removes the last BRCT domain upon Cre-mediated incision [54]. Mice homozygous for *Brca1*^*Δ22-24*^ allele are not generated to analyze the embryonic development. Unlike the lethality of *Brca1*^*Δ5-6/Δ5-6*^ null ES cells, viable *Brca1*^*Δ22-24/Δ22-24*^ ES cells can be obtained by Cre-mediated incision in *Brca1*^*F22-24/Δ22-24*^ ES cells [55]. An allele that disrupt both CC and BRCT domains, *Brca1*^*ΔC*^, is recently generated [56]. Mice homozygous for this allele is also lethal, but embryonic development is not analyzed.

In *Brca1*^*Δ22-24/Δ22-24*^ ES cells, no truncated BRCA1ΔBRCT proteins can be detected [55]. In *Brca1*^*5382stop/−*^ mouse tumors, no truncated BRCA1ΔBRCT proteins can be detected either [50]. Similarly, no truncated BRCA1ΔC proteins can be found in *Brca1*^*ΔC/ΔC*^ cells [56]. In agreement with these observations in mouse cells, truncated BRCA1ΔBRCT proteins cannot be detected either in several human cancer cell lines with truncating mutations at BRCA1 BRCT domains [56, 57]. It has been reported that many BRCT domain mutations cause folding defects and proteasomal degradation of these truncated proteins [58]. Based on these observations, it has been proposed that these BRCT domain mutant alleles are equivalent to *Brca1* null alleles. However, the defects of *Brca1*^*1700T/1700T*^ and *Brca1*^*5382stop/5382stop*^ embryos are less severe than *Brca1* null embryos, suggesting that these BRCT domain mutant alleles are unlikely true *Brca1* null allele. It is possible that truncated BRCA1ΔBRCT proteins are still present at low levels in these cells [50, 53].

Instead of disrupting the BRCT domain structure, a point mutation at BRCA1 BRCT domains is generated in *Brca1*^*S1598F*^ allele to specifically abolish the phosphorylated protein binding pocket without affecting protein stability [41]. Surprisingly, mice homozygous for *Brca1*^*S1598F*^ allele is viable. Interestingly, *Brca1*^*S1598F/S1598F*^ ES cells are also viable but have deficiency in HR repair. These observations suggest that HR deficiency can be tolerated in embryonic development in certain situations. Therefore, the overall structure, but not the phosphorylated protein binding abilities, of the tandem BRCT domains is important for the function of BRCA1 in embryonic development.

### Mice with disrupted phosphorylation

After DNA damage, BRCA1 is phosphorylated at serine 988 by CHK2 and is phosphorylated at serine 1189 by ATM [59, 60]. Since BRCA1 is required for certain ATM and ATR signaling, it is possible that these phosphorylation sites are important for BRCA1’s function. To address this possibility, *Brca1*^*S971A*^ and *Brca1*^*S1152A*^ alleles are generated in mice by mutating the amino acids corresponding to the phosphorylation sites in human BRCA1 [61, 62]. Mice homozygous for both alleles are viable and have no major developmental defects. Therefore, CHK2 and ATM-dependent phosphorylation of BRCA1 is dispensable for embryonic development.

## Rescue of embryonic development defects of *Brca1* mutant mice

Studies of *Brca1* mutant mice have revealed that BRCA1 is essential for embryonic development, which requires most of its domains including the RING domain, the BRCT domains, and the regions encoded by exon 11. Further studies have revealed that inactivating p53 signaling or 53BP1 can rescue the lethality or prolong the survival of *Brca1* mutant embryos, shedding lights on the mechanism of BRCA1’s functions in embryonic development. In this section, we will summarize our current understanding about how embryonic development of some *Brca1* mutant embryos can be rescued or prolonged and discuss the underlying mechanisms. A summary of these mutant alleles can be found in Table 2.

**Table 2:** Summary of *Brca1* mutant embryos with rescued survival or prolonged development after additional gene disruption

Brca1 mutant allele (homozygous)	Additional gene disruption	Embryonic development phenotypes	References
Rescue of embryonic development defects by compromising p53 signaling			
* Brca1*^*Δ5-6*^	*p53*^*−/−*^	Prolong the survival of embryo from E7.5 to E9.5	[63]
* Brca1*^*Δ5-6*^	*p21*^*−/−*^	Prolong the survival of embryo from E7.5 to E9.5	[63]
* Brca1*^*ex2*^	*p53*^*−/−*^	Improve the morphology of embryos at E8.5 and E9.5	[48]
* Brca1*^*11−*^	*p53*^*−/−*^	Extend the survival for 2 days	[42]
* Brca1*^*Δ223-763*^	*p53*^*−/−*^	Partially viable	[64]
* Brca1*^*Δ11*^	*p53*^+/−^ or *p53*^*−/−*^	Fully viable	[45]
* Brca1*^*Δ11*^	*Chk2*^+/−^*or Atm*^+/−^	Partially viable	[67]
* Brca1*^*Δ11*^	*Chk2*^*−/−*^* or Atm*^*−/−*^	Fully viable	[67]
Rescue of embryonic lethality by 53bp1 KO			
* Brca1*^*Δ11*^	*H2ax*^*−/−*^* or Rnf8*^*−/−*^* or Rnf168*^*−/−*^	Embryonic lethal, fail to rescue	[69, 80]
* Brca1*^*Δ11*^	*53bp1*^*−/−*^	Fully viable	[69]
* Brca1*^*Δ11*^	*53bp1*^*S25A/S25A*^	Fully viable	[83]
* Brca1*^*ex2*^	*53bp1*^*−/−*^	Fully viable	[71, 72]
* Brca1*^*ex2*^	*Rnf168*^*−/−*^	Fully viable	[80]
* Brca1*^*ΔC*^	*53bp1*^*−/−*^	Fully viable	[56]
* Brca1*^*Δ5-13*^	*53bp1*^*−/−*^	Partially viable	[82]

### Rescue of embryonic development defects by compromising p53 signaling

In most *Brca1* mutant mice, embryonic lethality is caused by cell death in postimplantation embryos. Interestingly, the death of *Brca1*^*Δ5-6/Δ5-6*^ embryos before E7.5 is preceded by a dramatic increase of p21 expression in E4 *Brca1*^*Δ5-6/Δ5-6*^ embryos [35]. p21 is an important cell cycle regulator whose activation leads to G1/S arrest. Since p21 is downstream of p53, a master controller of cell cycle arrest and apoptosis, it is likely that p53 signaling is activated in *Brca1*^*Δ5-6/Δ5-6*^ embryos. To test if p53 signaling activation is responsible for the lethality of *Brca1*^*Δ5-6/Δ5-6*^ embryos, *Brca1*^*Δ5-6/Δ5-6*^*;p53*^*−/−*^ and *Brca1*^*Δ5-6/Δ5-6*^*;p21*^*−/−*^ double mutant mice are generated. Although *p21* KO or *p53* KO fails to rescue the lethality of *Brca1*^*Δ5-6/Δ5-6*^ embryos, they can prolong the survival of these embryo from E7.5 to around E9.5 [63]. Similarly, *p53* KO can improve the morphology of *Brca1*^*ex2/ex2*^ embryos at E8.5 and E9.5 [48].

Mutant mice with different *Brca1* exon 11 disruptions have different phenotypes and most die at different embryonic stages. In some mutant mice, it has been examined if *p53* KO can rescue the embryonic lethality. In *Brca1*^*11−/−*^ embryos, which die between E7.5 and E9.5, *p53* KO can extend the survival for 2 days [42]. *Brca1*^*Δ223-763/Δ223-763*^ embryos die between E8.5 and E13.5, and the lethality can be significantly rescued by *p53* KO so that occasional viable *Brca1*^*Δ223-763/Δ223-763*^*;p53*^*−/−*^ double mutant mice can be obtained [64]. Most significantly, the embryonic lethality of *Brca1*^*Δ11/Δ11*^ embryos, most of which die between E12.5–18.5, can be fully rescued by *p53* heterozygosity or KO [45]. These observations suggest that p53 signaling activation contributes to the death of *Brca1* mutant embryos.

p53 activation is usually accompanied by its phosphorylation. After DSB formation, p53 can be phosphorylated by ATM at serine 15. ATM also phosphorylates CHK2, which can in turn phosphorylate p53 at serine 20 [65, 66]. These findings support that ATM-CHK2 signaling is important for p53 activation. Interestingly, the embryonic lethality of *Brca1*^*Δ11/Δ11*^ embryos can be fully rescued by *Chk2* KO or *Atm* KO or partially rescued by *Chk2* or *Atm* heterozygosity [67]. In line with these observations, *Chk2* KO can rescue the T cell development defects of T-cell specific *Brca1* knockout mice using *Brca1*^*flox5-6*^ mice [68]. Therefore, BRCA1 deficiency activates ATM-CHK2-p53 signaling, which plays a significant role in the death of *Brca1* mutant embryos.

### Rescue of embryonic lethality by *53bp1* KO

Studies have shown that *Brca1*^*Δ11/Δ11*^ mouse embryonic fibroblasts (MEFs) become senescent rapidly in culture, which can be suppressed by *p53* KO [45]. By screening factors required for premature senescence of *Brca1*^*Δ11/Δ11*^ MEFs, 53BP1 is identified among proteins involved in DNA damage response and cell cycle regulation [69]. *53bp1* KO not only suppresses premature senescence of *Brca1*^*Δ11/Δ11*^ MEFs, but also fully rescues the embryonic lethality of *Brca1*^*Δ11/Δ11*^ mice [69]. Interestingly, ATM-CHK2-p53 signaling is intact in *Brca1*^*Δ11/Δ11*^*;53bp1*^*−/−*^ cells, suggesting that the rescue is through a distinct mechanism. Subsequent study reveals that *53bp1* KO restores HR efficiency in *Brca1*^*Δ11/Δ11*^ cells [70]. Similarly, *53bp1* KO rescues the embryonic lethality of *Brca1*^*ex2/ex2*^ mice by restoring HR efficiency in *Brca1*^*ex2/ex2*^ cells [71, 72]. In line with these observations, 53BP1 loss rescues PARPi sensitivity of human *BRCA1* mutant cancer cells and contributes to PARPi resistance in *Brca1* null mouse breast cancer models [73]. Subsequent studies have revealed that loss of proteins associated with 53BP1, such as PTIP, RIF1, DYNLL1, and the Shielding complex, can also rescue the HR repair defect and PARPi sensitivity of human *BRCA1* mutant cancer cells [74–78]. It will be interesting to examine if loss of these 53BP1-associated proteins can promote the embryonic development of *Brca1* mutant mice.

Mechanistic studies of the above observations have revealed a novel function of BRCA1 in HR: regulating DSB repair pathway choice [13–15]. BRCA1 counteracts 53BP1’s block at DSB ends, promotes CTIP and MRE11-dependent DNA end resection, and directs DSB repair pathway choice towards HR. In cells with mutant BRCA1, such as *Brca1*^*Δ11/Δ11*^ and *Brca1*^*ex2/ex2*^ cells, 53BP1 remains at DSB ends, blocks DNA end resection, directs DSB repair pathway choice towards NHEJ, and causes HR deficiency. Loss of 53BP1 in *Brca1* mutant cells removes the block at DSB ends, allows DNA end resection to occur, and restores HR repair without intact BRCA1.

H2AX-MDC1-RNF8-RNF168 signaling pathways regulate histone ubiquitination upstream of 53BP1 in DNA damage response and are required for the recruitment of 53BP1 to DSB sites [79]. However, *H2ax* KO, *Rnf8* KO, or *Rnf168* KO fails to rescue the embryonic lethality of *Brca1*^*Δ11/Δ11*^ mice [69, 80]. On the contrary, *Rnf168* KO can rescue the embryonic lethality of *Brca1*^*ex2/ex2*^ mice [80]. Subsequent analyses reveal that the ability to interact with PALB2 is compromised in BRCA1Δ11 protein (encoded by *Brca1*^*Δ11*^ allele) but is maintained in BRCA1ΔRING protein (encoded by *Brca1*^*Δ2*^ allele) [80]. In addition to promoting histone ubiquitination, RNF168 also directly interacts with PALB2 and loads PALB2 to DSB sites [81], which serves as a backup mechanism for BRCA1-dependent PALB2 loading [80]. *Rnf168* KO prevents 53BP1’s block at DSB sites, restores DNA end resection, and directs DSB repair pathway choice towards HR in both *Brca1*^*Δ11/Δ11*^ and *Brca1*^*ex2/ex2*^ cells. However, due to BRCA1Δ11 protein’s defect in PALB2 interaction and loading, loss of RNF168-dependent PALB2 loading compromises overall PALB2 loading in *Brca1*^*Δ11/Δ11*^ cells and fails to rescue HR defects in these cells. On the contrary, since BRCA1ΔRING protein can interact and load PALB2, the loss of RNF168-dependent PALB2 loading has no impact on overall PALB2 loading in *Brca1*^*ex2/ex2*^ cells and HR is restored in these cells [80].

Although it is generally believed that HR deficiency is the major cause for embryonic lethality of *Brca1* mutant mice and *53bp1* KO rescues embryonic lethality of these mice by restoring HR, a recent study has challenged this idea by showing that *53bp1* KO rescues the embryonic lethality of *Brca1*^*ΔC/ΔC*^ mice without significantly restoring HR in *Brca1*^*ΔC/ΔC*^ cells [56]. BRCA1ΔC protein lacks the CC domain to interact with PALB2 and lacks the BRCT domains to locate to DSB site. In addition, BRCA1ΔC protein is not stable enough to be detected, making *Brca1*^*ΔC/ΔC*^ mice close to *Brca1* null mice. Therefore, despite rescuing DNA end resection by *53bp1* KO, PALB2 and BRCA2/RAD51 complex fails to be efficiently recruited to DSB sites, causing HR deficiency in *Brca1*^*ΔC/ΔC*^;*53bp1*^*−/−*^ cells. In agreement with this study, our recent study has also found that *53bp1* KO partially rescues the embryonic lethality of complete *Brca1* null mice (*Brca1*^*Δ5-13/Δ5-13*^) without restoring HR in complete *Brca1* null cells [82]. Similar observations have been made in a recent study that mutating 53BP1 to disrupt PTIP binding in *53bp1*^*S25A/S25A*^ mice can rescue the embryonic lethality of *Brca1*^*Δ11/Δ11*^ mice without significantly restoring HR in *Brca1*^*Δ11/Δ11*^ cells [83]. It is noteworthy that although largely HR deficient, minor restoration of HR is still observed in *Brca1*^*ΔC/ΔC*^;*53bp1*^*−/−*^ and *Brca1*^*Δ5-13/Δ5-13*^;*53bp1*^*−/−*^ cells, which is likely due to BRCA1-independent HR, such as RNF168-dependent PALB2 loading and HR. It is possible that such minor restoration of HR is sufficient for supporting embryonic development. Nevertheless, these studies suggest that HR deficiency might not be the major cause for embryonic lethality of *Brca1* mutant mice.

BRCA1 is important for protecting replication fork from collapsing after replication stress [20]. However, although *53bp1* KO can rescue the lethality of *Brca1*^*Δ11/Δ11*^, *Brca1*^*ex2/ex2*^, and *Brca1*^*ΔC/ΔC*^ embryos, it cannot rescue the replication fork protection defects in *Brca1*^*Δ11/Δ11*^, *Brca1*^*ex2/ex2*^, or *Brca1*^*ΔC/ΔC*^ cells [20, 56, 72]. Therefore, replication fork protection defect unlikely contributes significantly to the embryonic lethality of *Brca1* mutant mice. The major cause for the embryonic lethality of *Brca1* mutant remains to be clarified.

## Conclusions

As a key protein that promotes DSB repair by HR, BRCA1 has been extensively studied using multiple approaches. Since the first report of *Brca1* mutant mice more than 20 years ago, many different *Brca1* mutant mice have been generated to study the physiological functions of BRCA1 in vivo. Among various defects identified in *Brca1* mutant and conditional mutant mice, embryonic lethality remains the most significant phenotypes of most *Brca1* mutant mice. Analyses of embryos and cells from these mice have not only complemented the in vitro findings that BRCA1 is important for HR, but also clarified the impact of different domain deletions and mutations on HR. Importantly, studies of gene disruptions that rescue the embryonic lethality of *Brca1* mutant mice have led to the discovery of a novel function of BRCA1 in DSB repair pathway choice. Collectively, studies of embryonic development of *Brca1* mutant mice have significantly advanced our understanding of BRCA1’s functions in HR. Generating additional *Brca1* mutant mouse models in future can facilitate addressing unsolved questions about BRCA1’s functions in HR.

## Data Availability

Not applicable.
